# Role of Concentration in Opposing Effects of Anandamide on Nociceptive Synapses versus Non-nociceptive Synapses

**DOI:** 10.1523/ENEURO.0480-25.2026

**Published:** 2026-04-30

**Authors:** Brian D. Burrell

**Affiliations:** Division of Biomedical and Translational Sciences, Center for Brain and Behavior Research, Sanford School of Medicine, University of South Dakota, Vermillion, South Dakota 57069

**Keywords:** anandamide, endocannabinoid, *Hirudo*, nociception, synaptic plasticity

## Abstract

There is considerable interest in cannabinoid-based therapies to treat pain, but activation of the endogenous cannabinoid (endocannabinoid) system can elicit pro- and anti-nociceptive effects. This study tests the hypothesis that the concentration of the endocannabinoid arachidonoylethanolamine (AEA) contributes to whether pro- or anti-nociceptive effects are observed. Experiments were carried out using isolated ganglia from the medicinal leech *Hirudo verbana* where it is possible to selectively record from nociceptive and non-nociceptive synapses in the central nervous system (CNS). Previous studies using *Hirudo* have shown that endocannabinoids depress nociceptive (N) sensory cell synapses and potentiate of non-nociceptive pressure (P) sensory cell synapses. In this study, exogenously applied AEA produced depression of N synapses and potentiation of P synapses across the same range of concentrations. However, the results differed when using URB597, a drug that raises AEA by inhibiting fatty acid amine hydrolase (FAAH), the enzyme that metabolizes AEA. Potentiation of P synapses required higher concentrations of URB597 compared with the concentrations needed to elicit depression of N synapses. Interestingly, pairing somatosensory afferent activity with a normally subthreshold concentration of URB597 did elicit potentiation in P synapses. Sensitivity of the nociceptive and non-nociceptive synapses to cannabinoid receptor inhibitors differed when AEA versus UBR597 was applied. This study demonstrates the complexity of AEA-mediated effects on distinct synapse types that may be informative about the basic biology of endocannabinoid modulation of nociception.

## Significance Statement

While there is considerable interest in developing cannabinoid-based analgesics, activation of the endocannabinoid system can produce pro- and anti-nociceptive effects, which are produced may be due to the endocannabinoid concentration. This concentration hypothesis was examined by assessing the effects of arachidonoylethanolamine (AEA) on identifiable nociceptive and non-nociceptive synapses in *Hirudo verbana* (the medicinal leech). Both types of synapses were equally sensitive to exogenously applied AEA, but when endogenous AEA levels were increased using inhibitors of AEA metabolism, nociceptive synapses were much more sensitive compared with non-nociceptive synapses. These findings may inform why past cannabinoid-based therapies have failed as analgesics and how to develop effective analgesic therapies in the future.

## Introduction

Endogenous cannabinoids (endocannabinoids) are signaling lipids that are synthesized and released in an activity-dependent manner. The two main endocannabinoids are N-arachidonoylethanolamine (AEA) and 2-arachidonoylglycerol (2-AG). AEA is primarily synthesized by NAPE-phospholipase D (NAPE-PLD) and metabolized by fatty acid amide hydrolase (FAAH), whereas the corresponding enzymes for 2-AG are diacylglycerol lipase (DAGL) and monoacylglycerol lipase (MAGL; Lu and Mackie, 2016; Duncan et al., 2024). Both AEA and 2-AG bind to the metabotropic cannabinoid receptors (CB1 and CB2) and the transient potential vanilloid 1 channel (TRPV1; Zygmunt et al., 1999, 2013). While there is considerable interest in developing cannabinoid-based analgesics, clear evidence that these therapies are effective is lacking (De Vita et al., 2018; Fisher et al., 2020; Mun et al., 2020; Finn et al., 2021; Hsu et al., 2025). A critical issue, therefore, is better understanding the basic biology of endocannabinoid modulation of somatosensory signaling.

One of the most prominent effects of endocannabinoids is depression of synaptic transmission (Lu and Mackie, 2016). Endocannabinoids elicit long-term depression (eCB-LTD) in synapses of primary nociceptive afferents, which likely produces an analgesic effect (Kato et al., 2012). However, endocannabinoids also depress inhibitory synapses leading to disinhibition, and endocannabinoid-mediated disinhibition of spinal somatosensory circuits can produce pro-nociceptive effects (Pernia-Andrade et al., 2009).

**Figure 1.**
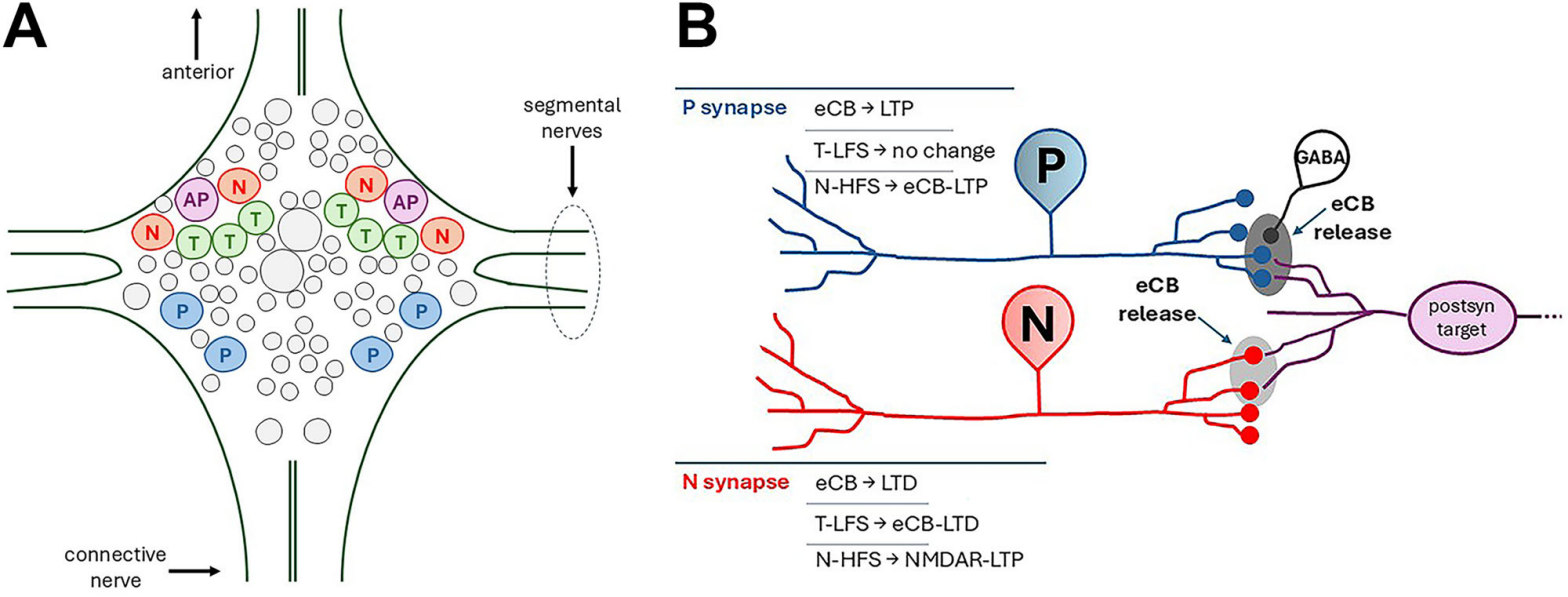
Overview of the *Hirudo* CNS and synaptic pathways. ***A***, Diagram of a single *Hirudo* ganglion showing the T (touch), P (pressure), and N (nociceptive) somatosensory neurons and the AP (anterior pagoda) cell which is the postsynaptic target of all three afferents. The connective nerves link each ganglion to its neighboring anterior and posterior ganglia. The segmental nerves connect the CNS to the periphery and both nerves contain sensory and motor neuron axons. ***B***, Circuit diagram of the P and N synapses that converge onto a shared postsynaptic target (data summarized are from both N/P-to-AP and -to-L motor neuron experiments). P synapses undergo tonic inhibition due to GABAergic interneurons (GABA). Endocannabinoids depress this tonic inhibition of the P synapse, disinhibiting the synapses and gating LTP. In the case of N synapses, endocannabinoids directly depress excitatory (glutamatergic) transmission onto the postsynaptic targets. Gray ovals around the N, P, and GABA presynaptic terminals represent the hypothetical mechanism in which low amounts of endocannabinoids (light gray) elicit eCB-LTD in N synapses and higher levels (dark gray) elicit eCB-LTP in P synapses.

Studies using the medicinal leech, *Hirudo verbana*, have contributed to a better understanding the pro- and anti-nociceptive functions of the endocannabinoid system (Paulsen and Burrell, 2019). The endocannabinoid system is well-conserved throughout the animal kingdom with 2-AG and AEA observed from pre-bilaterian invertebrates to humans (Elphick, 2012). In *Hirudo*, AEA and 2-AG are present in the CNS, and *Hirudo* versions of FAAH and MAGL have been characterized (Matias et al., 2001; Kabeiseman et al., 2020, 2024). The *Hirudo* CNS consists of 21 ganglia linked in a chain by a connective nerve with each ganglion having two bilateral pairs of segmental nerves projecting to the periphery (Fig. 1*A*; Muller et al., 1981). Each ganglion has ≅400 neurons, many of which are readily identifiable based on size, position, and electrophysiological characteristics. This permits detailed electrophysiological studies of neurons and their synapses, linking neurophysiological properties with behavioral functions (Kristan et al., 2005). Similar to mammalian somatosensory system, *Hirudo* have fast-adapting touch-sensitive (T) neurons, slow-adapting pressure-sensitive (P) neurons, and mechanical and polymodal nociceptive (N) neurons (Nicholls and Baylor, 1968; Carlton and McVean, 1995; Pastor et al., 1996; Smith and Lewin, 2009; Summers et al., 2014).

Pro- and anti-nociceptive endocannabinoid effects reported in mammals have also been observed in *Hirudo* somatosensory synapses (summarized in Fig. 1*B*) and at the behavioral level. In terms of anti-nociceptive effects, 2-AG and AEA depressed N synapses and reduced withdrawal responses elicited by N cell activity (Yuan and Burrell, 2012; Wang and Burrell, 2016; Summers et al., 2017). Furthermore low-frequency stimulation (LFS) of the non-nociceptive T afferents elicited heterosynaptic eCB-LTD in N synapses and a reduction in nociceptor-elicited withdrawal reflexes (Yuan and Burrell, 2010, 2013; Hanson and Burrell, 2018). This has also been observed in rodents, i.e., repetitive stimulation of Aβ fibers elicited eCB-LTD in C fiber synapses and depression of nocifensive reflexes (Yang et al., 2016). In terms of pro-nociceptive effects, 2-AG and AEA potentiate the non-nociceptive P cell synapses via depression of tonic GABAergic input leading to disinhibition (Higgins et al., 2013; Wang and Burrell, 2016; Paulsen and Burrell, 2022; Franzen et al., 2023). Endocannabinoid-mediated potentiation of P synapses was also produced by high-frequency stimulation (HFS) of N cells. Critically, both endocannabinoid-mediated depression of N synapses and potentiation of P synapses have been linked to decreases and increases, respectively, of the *Hirudo* withdrawal reflex in semi-intact preparations where the same N or P cell used in synaptic measurements was also used to elicit behavior (Yuan and Burrell, 2013; Wang and Burrell, 2018).

It has been suggested that pro- and anti-nociceptive effects of endocannabinoids are concentration dependent, e.g., lower concentrations being analgesic and higher concentrations pro-algesic (Morisset et al., 2001; Bhattacharyya et al., 2025). In the present study, this concentration hypothesis was assessed by examining the AEA concentrations that produce depression in *Hirudo* N synapses versus potentiation in P synapses. The effects of increasing AEA levels endogenously using the FAAH inhibitor URB597 were also examined as well as the effects of CB1 and TRPV1 inhibitors on AEA- and URB597-mediated synaptic plasticity.

## Materials and Methods

### General

*Hirudo verbana* weighing 2–3 g (North America BioPharma) were maintained in artificial pond water (0.5 g/L Instant Ocean Sea Salt) at 15°C in a refrigerated incubator with a 12 h light/dark cycle in. Electrophysiological recordings were carried out in *Hirudo* saline (in mM: 110 NaCl, 4 KCl, 1.8 CaCl_2_, 1 MgCl_2_, 5 NaOH, 10 glucose, and 10 HEPES, pH 7.4). *Hirudo* are sanguineous and can survive 12 months or longer on a single blood meal (Dickinson and Lent, 1984); however, *Hirudo* for this project were fed every 6 months with commercially available bovine blood (Hemostat Laboratories). Because they are invertebrates, *Hirudo* are not subject to formal experimental animal use and care regulations. Nevertheless, animals were anesthetized by immersion in a 50 ml container filled with cold (4°C) high magnesium saline (*Hirudo* saline + 15 mM MgCl_2_) with 5% ethanol for 45–60 min after which leeches were flaccid and immobile. In addition, dissections were carried out in a dish filled with ice-cold high magnesium saline which reduces neuro-signaling. For each experiment a single mid-body ganglion (segments 7–18) was removed and pinned onto Sylgard-lined 35 mm petri dish with a fluid exchange insert and filled with *Hirudo* saline (chamber volume ≈650 µl). There was constant perfusion of the recording chamber at a rate of ≈1.5 ml/min, and ganglia were held for at least 15 min before recordings began to wash out the effects of the high magnesium saline used during dissection. Ganglia were not desheathed for electrophysiological recordings.

### Electrophysiology

Current-clamp recordings were made using an Axoclamp 900A amplifier set on bridge mode with signals digitally converted for analysis using a Digidata 1440A or 1550B interface (Molecular Devices). Sharp intracellular microelectrodes were fashioned from borosilicate glass (0.78 mm ID, 1 mm OD) using a P-97 Flaming/Brown micropipette puller (Sutter Instrument). Electrodes were filled with 2 M KAc and had a tip resistance of 25–35 MΩ. Current-clamp recordings and current injection into neurons were carried out using pClamp 10.1.

In each experiment dual intracellular recordings were made from the anterior pagoda (AP) cell, which served as the postsynaptic neuron, and either the nociceptive (N) cell or pressure-sensitive (P) cell as the presynaptic neurons (Fig. 1*A*). The AP receives glutamatergic inputs from both the P and N cells (Fig. 1*B*) and may be part of an arousal neural circuit in the *Hirudo* CNS (Frady et al., 2016). In a subset of experiments, intracellular recordings of touch-sensitive (T) cells were made for the purpose of delivering T-cell low-frequency stimulation (T-LFS) which has previously been shown to elicit eCB-LTD in N synapses (Yuan and Burrell, 2010). Neurons were visualized using a stereo microscope, and dark-field illumination with T, P, N, and AP neurons was identified by their size, characteristic position within the ganglion, and action potential shape (Muller et al., 1981).

Each synapse experiment had a pretest and posttest recording, both consisting of tests of excitatory postsynaptic potential (EPSP) amplitude, paired-pulse facilitation, and postsynaptic input resistance. At the end of the pretest recording, the intracellular electrodes were removed from the pre- and postsynaptic neurons and the treatment stage immediately followed (drug treatment or T-cell LFS). Because the goal of these experiments was to examine changes in signaling that persisted beyond the immediate treatment, posttest recordings of synapse were not made until 30 min after the treatment ended, at which point the electrodes were re-inserted into the pre- and postsynaptic neurons. The electrodes were not kept in the neurons for the full 45 min duration of the experiment because this leads to cell damage due to osmotic stress.

**Figure 2.**
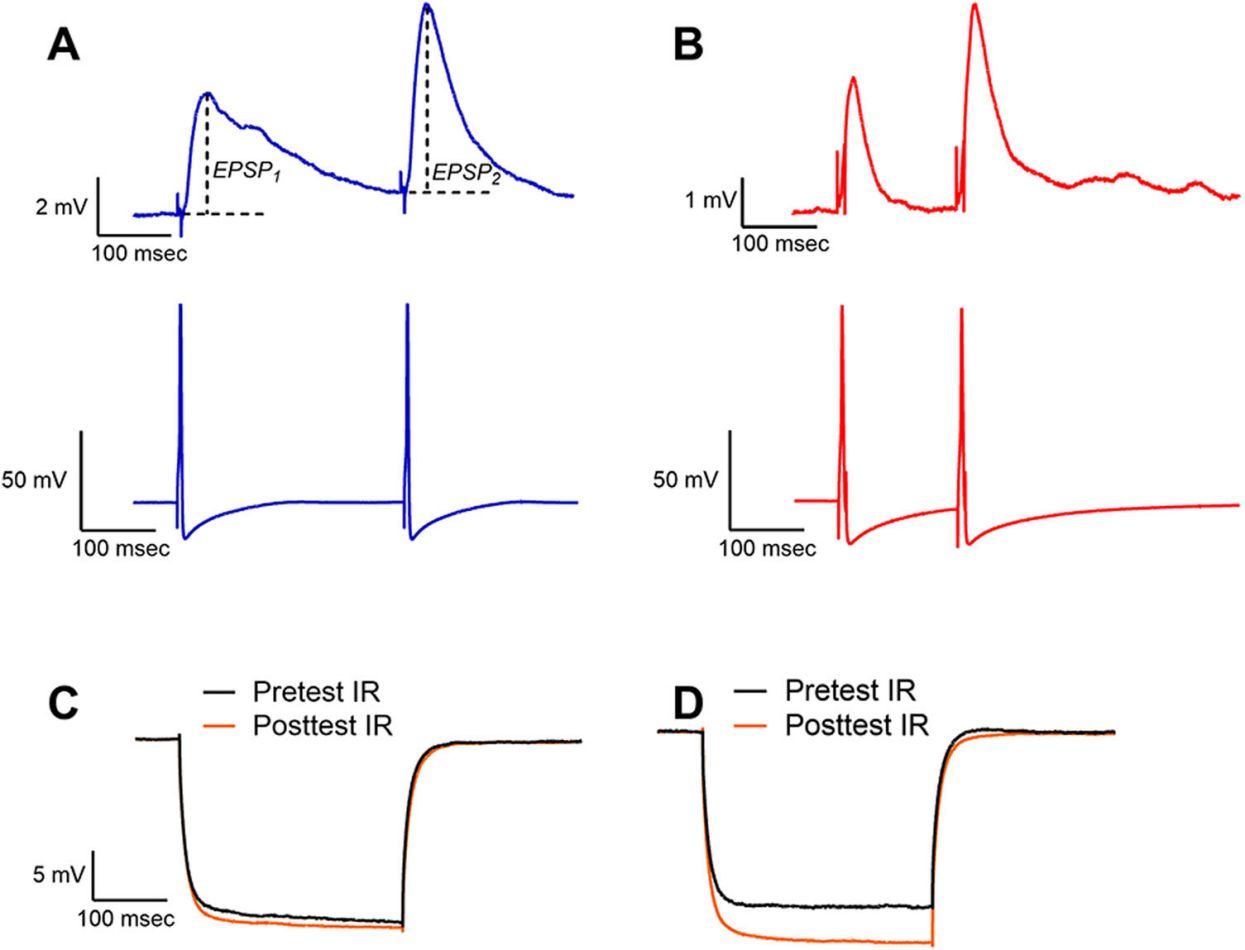
Examples of PPR and input resistance recordings. ***A***, Paired EPSPs (top traces) elicited by two P cell action potential (bottom traces). EPSP_1_ and EPSP_2_ represent amplitudes of the first and second EPSP, respectively. ***B***, Paired EPSPs (top traces) elicited by two N cell action potential (bottom traces). Interval between twin N action potentials was shorter compared with P cell since the N-AP synapse EPSP decays more quickly. ***C***, Example of pretest (black) and posttest (red) input resistance (IR) traces from an experiment with minimal change in input resistance. ***D***, Example of pair of traces where there was a >15% change in IR. Experiments exhibiting such changes in IR between the pre- and posttest were excluded from analysis.

EPSPs in the AP cell were elicited by injecting depolarizing current pulses (3–4 nA, 5 ms) into the presynaptic neurons (either P or N cells). Twin presynaptic action potentials were elicited resulting in two EPSPs recorded in the AP cell (EPSP_1_ and EPSP_2_; Fig. 2*A*,*B*). The interval between the paired EPSPs was 150 ms for N-to-AP (N-AP) synapses and 300 ms for the P-to-AP (P-AP) synapses which had slower EPSP decay rate. EPSP_1_ in the pre- and posttest was used to measure treatment-induced changes in EPSP amplitude (in mV). The ratio of the 2nd and 1st EPSP amplitudes, i.e., the paired-pulse ratio or PPR (EPSP_2_/EPSP_1_), was used to measure paired-pulse facilitation, a form of short-term synaptic plasticity that can indicate whether changes in synaptic transmission have a pre- or postsynaptic locus (Dobrunz and Stevens, 1997; Zucker and Regehr, 2002). These EPSP recordings were made every 20 s and alternated with tests of postsynaptic input resistance (IR; 10 s between each EPSP and IR test). IR was tested by injecting negative current into the AP cell (−1 nA, 300 ms) and measuring the maximum change in voltage. Measurements of pre- and posttreatment IR were compared to assess the recording quality of the postsynaptic cell and also provided a test of whether changes in the neuron's intrinsic electrical properties contributed to changes in EPSP amplitude. An example of stable IR recordings is shown in Figure 2*C*. Experiments in which the postsynaptic IR increased or decreased by >15% between the pretest and posttest recordings were discarded (Fig. 2*D*). This ±15% range was chosen as being sufficiently sensitive to the potential damage of the AP cell due to the two separate penetrations with the intracellular electrode, while still allowing for detection of potential changes in IR that might contribute to treatment-induced increases or decreases in EPSP amplitude. Across all the experiments carried out, an average of 0.64 experiments per treatment group (e.g., pharmacological concentration or T-LFS group) was excluded based on this IR-based quality control criteria. During all recordings the membrane potential of the AP cell was hyperpolarized to approximately −80 mV to prevent postsynaptic action potentials from interfering with EPSP measurements. Activity-dependent LTD in P-AP or N-AP synapses was elicited via T-LFS consisting of 900 current pulses with a 10 ms duration, 3 nA amplitude, and delivered at 1 Hz.

### Pharmacological treatments

All drugs were stored in frozen aliquots and diluted to the desired concentration with *Hirudo* normal saline immediately before use. Stock solutions of arachidonoylethanolamine (AEA; Cayman Chemical Company or Sigma-Aldrich) were prepared in either ethanol (EtOH) for the concentration series experiments or dimethyl sulfoxide (DMSO) for pharmacology experiments. Separate vehicle control experiments using EtOH or DMSO dissolved in saline were carried out. Stock solutions of URB597 (FAAH inhibitor), SB366791 (TRPV1 inhibitor), and AM251 (CB1 reverse agonist; all from Tocris) were prepared using DMSO. For AEA-only or URB597 experiments, drugs were perfused through the recording chamber for 15 min following the completion of pretest EPSP recordings. For experiments that used a receptor inhibitor, the ganglion was pretreated with the inhibitor for 5 min, followed by delivery of the drug + inhibitor mixture for 15 min. Following the end of pharmacological treatments or T-LFS, the recording chamber was continuously perfused with *Hirudo* saline (washout) for 30 min followed by the posttest recording.

### Data analysis

A given treatment (drug or LFS) was only tested in one ganglion per animal; therefore, sample size represents the number of animals tested. In rare situations where the same experimental treatment was carried out twice in the same animal, the data was averaged from these two experiments. For each pre- and posttest, EPSP, PPR, and IR values were the average of 5–10 sweeps per recording. As previously stated, experiments were excluded from analysis if the IR changed by >15% between the pre- and posttest. Experiments were also excluded if the pretest EPSP amplitude is >7 mV since synapses that large often exhibit a “ceiling effect” in terms of being able to undergo further potentiation (Burrell and Li, 2008). For analysis purposes, EPSP data were converted to “% of initial EPSP” (100 * [EPSP_posttest_ / EPSP_pretest_]) so that values <100% indicate a decrease in EPSP amplitude and values >100% indicate an increase in EPSP size. A similar protocol was used for changes in IR (e.g., % initial IR = 100 * [IR_posttest_ / IR_pretest_]). Data were shown as mean ± standard error (SE) and statistical analysis and graphing carried out using GraphPad version 10.6.1 for Windows (GraphPad Software). Data normality was confirmed prior to using parametric statistics using quantile–quantile plots. Two-way and one-way analysis of variance (ANOVA) was used to assess effects of drug, LFS, and drug + LFS effects followed by either Tukey (one-way ANOVA) or Sidak (two-way ANOVA) tests for post hoc comparisons. Paired *t* tests were used to assess changes in PPR following AEA treatment.

## Results

**Figure 3.**
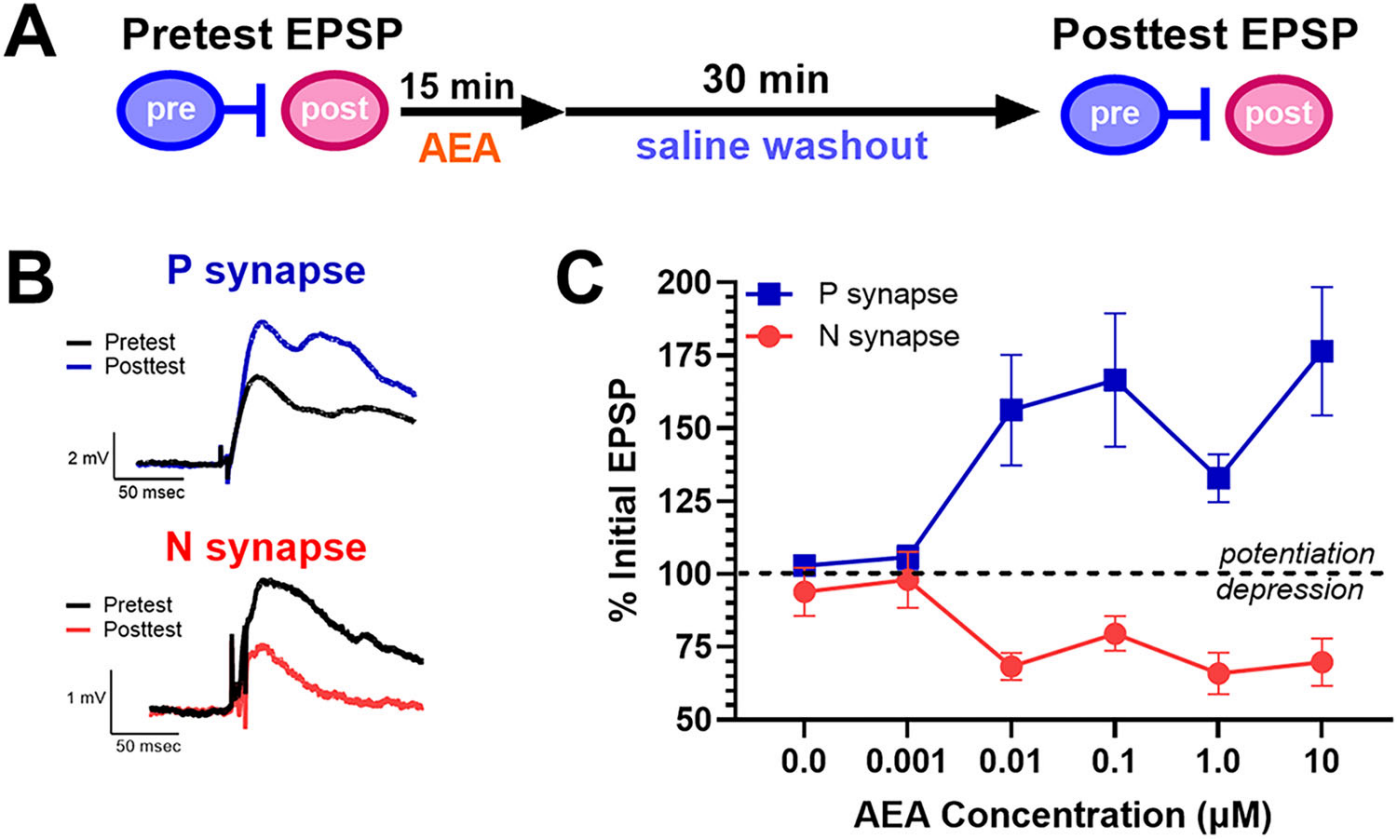
Concentration-dependent effects of AEA on P-AP versus N-AP synapses. ***A***, Timeline of experimental protocol. ***B***, Sample EPSP traces from the P-AP and N-AP synapses during pretest in saline and posttest following AEA treatment. ***C***, Concentration profile of the potentiating effects of AEA on P-AP synapses (blue square) and depression on N-AP synapses (red circles). Dashed line at 100% indicates no change in EPSP amplitude between the pre- and posttest. Sample size for each P synapse groups were 0 µM (0.001% EtOH vehicle) *n* = 5, 0.001 µM *n* = 8, 0.01 µM *n* = 8, 0.1 µM *n* = 8, 1.0 µM *n* = 10, and 10 µM *n* = 6. Sample size for each N synapse groups were 0 µM (vehicle) *n* = 7, 0.001 µM *n* = 6, 0.01 µM *n* = 8, 0.1 µM *n* = 9, 1.0 µM *n* = 8, and 10 µM *n* = 8. Linear curve fitting of the AEA concentration profiles for the P-AP and N-AP synapses is shown in Extended Data Figure 3-1. Effects of AEA concentrations on postsynaptic IR for both synapses are shown in Extended Data Figure 3-2.

Experiments were carried out in the P-AP or N-AP synapses. Synapses onto the AP neuron were chosen because this neuron is one of the first activated by mechanosensory input and may be part of an arousal network in *Hirudo* (Frady et al., 2016). Changes in P-AP or N-AP EPSP amplitude were measured following AEA treatment of 0, 0.001, 0.01, 0.1, 1.0, or 10 µM (Fig. 3*A*). AEA potentiated P-AP synapses and depressed N-AP synapses (Fig. 3*B*), and there were no differences in the concentration profiles of AEA for either synapse (Fig. 3*C*). A two-way ANOVA detected a significant effect of synapse type (P-AP vs N-AP; *F*_(1,79)_ = 74.13, *p* < 0.0001), no effect of concentration (*F*_(5,79)_ = 1.78, *p* = 0.13), but a significant synapse–concentration interaction effect (*F*_(5,79)_ = 5.54, *p* < 0.0005). Based on linear curve fitting, the EC_50_ values of AEA were nearly identical for the P-AP and N-AP synapses, 0.0020 and 0.0022 µM, respectively (Extended Data Fig. 3-1). No apparent changes in the input resistance (IR) of the postsynaptic AP cell were observed (Extended Data Fig. 3-2*A*). A one-way ANOVA did detect a significant difference across AEA concentrations in the P-AP synapses (*F*_(5,39)_ = 2.71, *p* < 0.05), but post hoc analysis showed that this was due exclusively to a single significant difference in IR between the 0.01 and 0.1 µM AEA groups (*p* < 0.05). No changes in postsynaptic IR were observed in the N-AP synapses as well (Extended Data Fig. 3-2*B*; *F*_(5,39)_ = 0.37). Together, these results indicate that the concentration of exogenously applied AEA did not determine whether depression of N-AP synapses or potentiation of P-AP synapses was produced.

10.1523/ENEURO.0480-25.2026.f3-1 Figure 3-1 Comparison of concentration profiles for AEA. (A) P-AP synapse. (B) N-AP synapse. Download Figure 3-1, TIF file.

10.1523/ENEURO.0480-25.2026.f3-2 Figure 3-2 Effects of AEA on postsynaptic (AP cell) input resistance (IR). Data is expressed as a percent of the initial pretest level (100[posttest IR/pretest IR]). Effects of increasing AEA concentrations from the P-AP (A) and N-AP (B) synapses. In the P-AP synapses, none of the AEA-treated synapses were different from the vehicle control group (0 μM), although there was a statistically significant difference in IR between the 0.01 and 0.1 μM groups (p<0.05). In the N-AP synapses, none of the AEA-treated synapses were different from the vehicle control group. Download Figure 3-2, TIF file.

**Figure 4.**
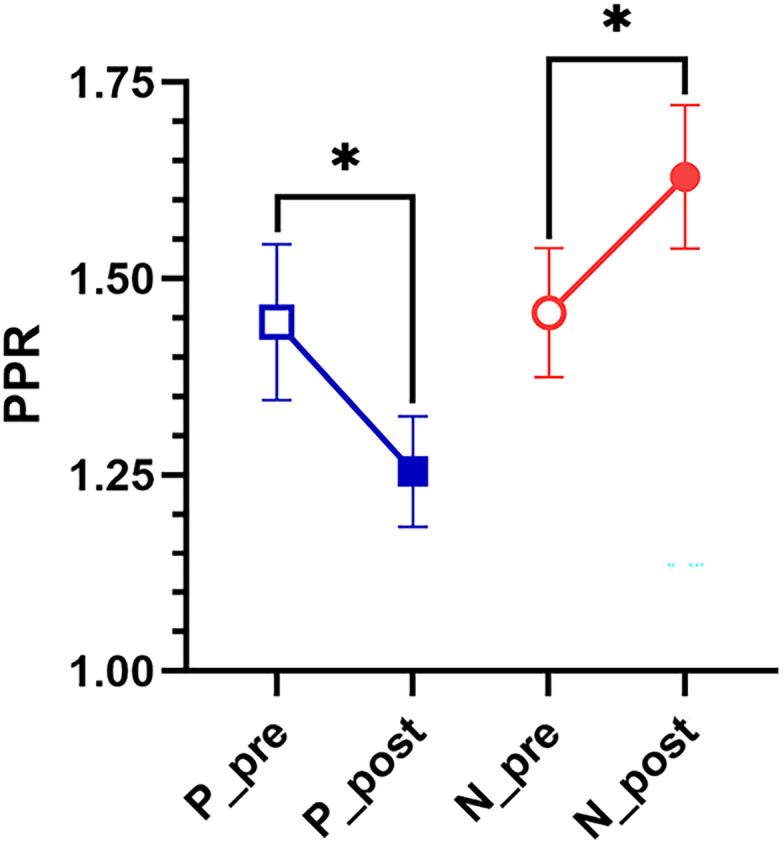
AEA-induced changes in PPR in P-AP and N-AP synapses. In P synapses (*n* = 29), the average PPR ratio following AEA treatments (filled blue square) was significantly lower compared with pretest levels in normal saline (open blue square). In N-AP synapses (*n* = 33), the average PPR ratio following AEA treatments (filled red circle) was significantly higher compared with pretest levels (open red circle).

For analysis of paired-pulse facilitation, since the level of AEA-induced potentiation or depression was relatively equivalent across the 0.1–10 µM concentrations, the PPRs from all these concentrations were combined for analysis. For AEA-induced potentiation of P-AP synapses, the PPR significantly decreased between the pre- and posttests (Fig. 4; paired *t*_(28)_ = 2.33, *p* > 0.05), suggesting that AEA-induced potentiation involves an increase in neurotransmitter release. For AEA-induced depression of N-AP synapses, the PPR significantly increased (Fig. 4; paired *t*_(32)_ = 2.66, *p* < 0.05), suggesting that AEA-induced depression involves a decrease in neurotransmitter release. These results suggest that AEA-induced effects on both synapses were mediated at the presynaptic level.

**Figure 5.**
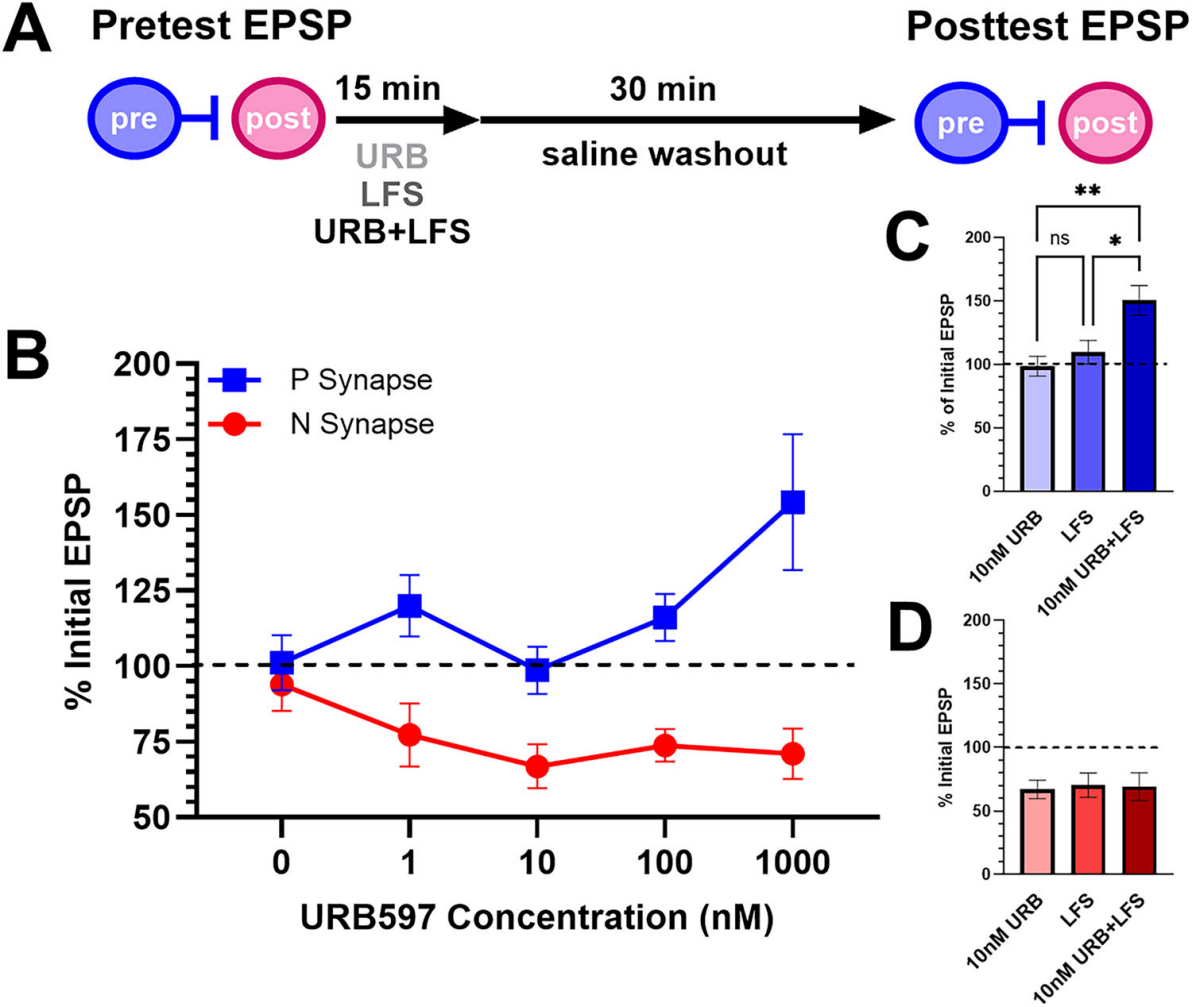
Effects of URB and T-LFS on P-AP and N-AP synapses. ***A***, Timeline of experimental protocol. Pretest recordings of the P-AP or N-AP synapses were followed by either 15 min treatment with URB597 alone, T-LFS (1 Hz for 15 min), or T-LFS + URB (also 15 min). Following a 30 min washout period, posttest recordings were carried out of either synapse. ***B***, A comparison of the effects of 0–1,000 nM URB597 on P-AP synapses (blue squares) versus N-AP synapses (red circles). Dashed line at 100% indicates no change in the posttest EPSP relative to the pretest amplitude. Sample size for each P-AP synapse groups were 0 nM (0.001% DMSO vehicle), *n* = 6, 1 nM *n* = 6, 10 nM *n* = 7, 100 nM *n* = 6, and 1,000 nM *n* = 6. Sample size for each N-AP synapse groups were 0 nM, *n* = 6, 1 nM *n* = 6, 10 nM *n* = 5, 100 nM *n* = 5, and 1,000 nM *n* = 8. ***C***, Bar graph of documenting the % of initial EPSP in the P-AP synapse for the URB (*n* = 7), LFS (*n* = 10), and URB + LFS (*n* = 12) treatment groups. ***D***, Bar graph of documenting the % of initial EPSP in the N-AP synapses for the URB (*n* = 5), LFS (*n* = 4), and URB + LFS (*n* = 5) treatment groups. * indicates *p* ≤ 0.05, ** indicates *p* ≤ 0.01. Effects of URB594 concentrations and T-LFS on postsynaptic IR for both synapses are shown in Extended Data Figure 5-1.

An alternative approach to exogenously applying AEA is to increase endogenous levels of AEA by inhibiting its metabolism by FAAH. *Hirudo* possess an ortholog of FAAH (hirFAAH) that hydrolyzes AEA via a catalytic triad that is conserved with vertebrate FAAH (Kabeiseman et al., 2024). URB597, a widely used and selective inhibitor of FAAH, has been validated for inhibiting hirFAAH hydrolase of AEA in a concentration-dependent manner (Kabeiseman et al., 2024). In the current study, the concentration-dependent effects of URB597 were examined in the P-AP and N-AP synapses (1 nM–1 µM URB597; Fig. 5*A*). URB597 did depress N-AP synapses and potentiate P-AP synapses, similar to the effects of AEA, but the concentration profile differed considerably between the two synapses. Only the highest concentration of URB597 produced substantial potentiation in P-AP synapses (Fig. 5*B*). N-AP synapses were depressed at all URB597 concentrations tested. A two-way ANOVA comparing the effects of URB597 concentrations and synapse type detected a significant effect of synapse type (*F*_(1,50)_ = 36.34, *p* < 0.0001), no significant effect of concentration (*F*_(4,50)_ = 2.03, *p* = 0.10), but a significant synapse–concentration interaction effect (*F*_(4,51)_ = 3.44, *p* < 0.05).

10.1523/ENEURO.0480-25.2026.f5-1 Figure 5-1 Effects of URB597 and T-LFS on postsynaptic IR. Increasing AEA concentrations are represented by solid fills. Effects of LFS and URB+LFS treatments are shown as diagonal fill patterns. None of these treatments exhibited an effect on input resistance in P-AP (A) and N-AP (B) synapses. Download Figure 5-1, TIF file.

The differences in the concentration-dependent effects of URB597 in the P-AP versus N-AP synapses suggest that the two synapses differ in their sensitivity to endogenously released AEA. To further test this idea, the ability of URB597 to modulate activity-induced synaptic plasticity was observed. In *Hirudo*, LFS of touch afferents (T-LFS) selectively elicits eCB-LTD in N synapses but does not alter P synapses (Wang and Burrell, 2016), possibly because T-LFS produces insufficient AEA to disinhibit/potentiate P synapses. Tests of P-AP synapses were carried out in three groups of ganglia (Fig. 5*A*), one where T-LFS alone was delivered, a second treated with 10 nM URB597, and a third in which T-LFS was delivered with 10 nM URB597 (T-LFS + URB). Neither T-LFS nor 10 nM URB597 alone altered P-AP EPSP amplitude, but T-LFS + URB597 did potentiate P-AP synapses (Fig. 5*C*). A one-way ANOVA confirmed a significant effect of treatment (*F*_(2,26)_ = 6.85, *p* < 0.005) with post hoc analysis showing a significant difference between the T-LFS + URB group compared with the T-LFS-alone and URB-alone groups (*p* < 0.05 and 0.01, respectively). These results suggest that T-LFS can be made to elicit potentiation in P-AP synapses when endogenous AEA levels are presumably boosted by the FAAH inhibitor.

For the N-AP synapses, both T-LFS and 10 nM URB597 produced similar levels of depression (Fig. 5*D*), indicating that a putative increase in endogenous AEA levels during T-LFS did not lead to greater eCB-LTD. A one-way ANOVA confirmed that there was no significant difference in N-AP synapse depression between the three groups (*F*_(2,11)_ = 0.04, *p* = 0.97). In terms of postsynaptic IR, no effects of URB597, T-LFS, or URB + LFS were observed in the P-AP and N-AP synapses (Extended Data Fig. 5-1; one-way ANOVA P-AP *F*_(6,48)_ = 0.21; N-AP *F*_(6,48)_ = 0.30).

**Figure 6.**
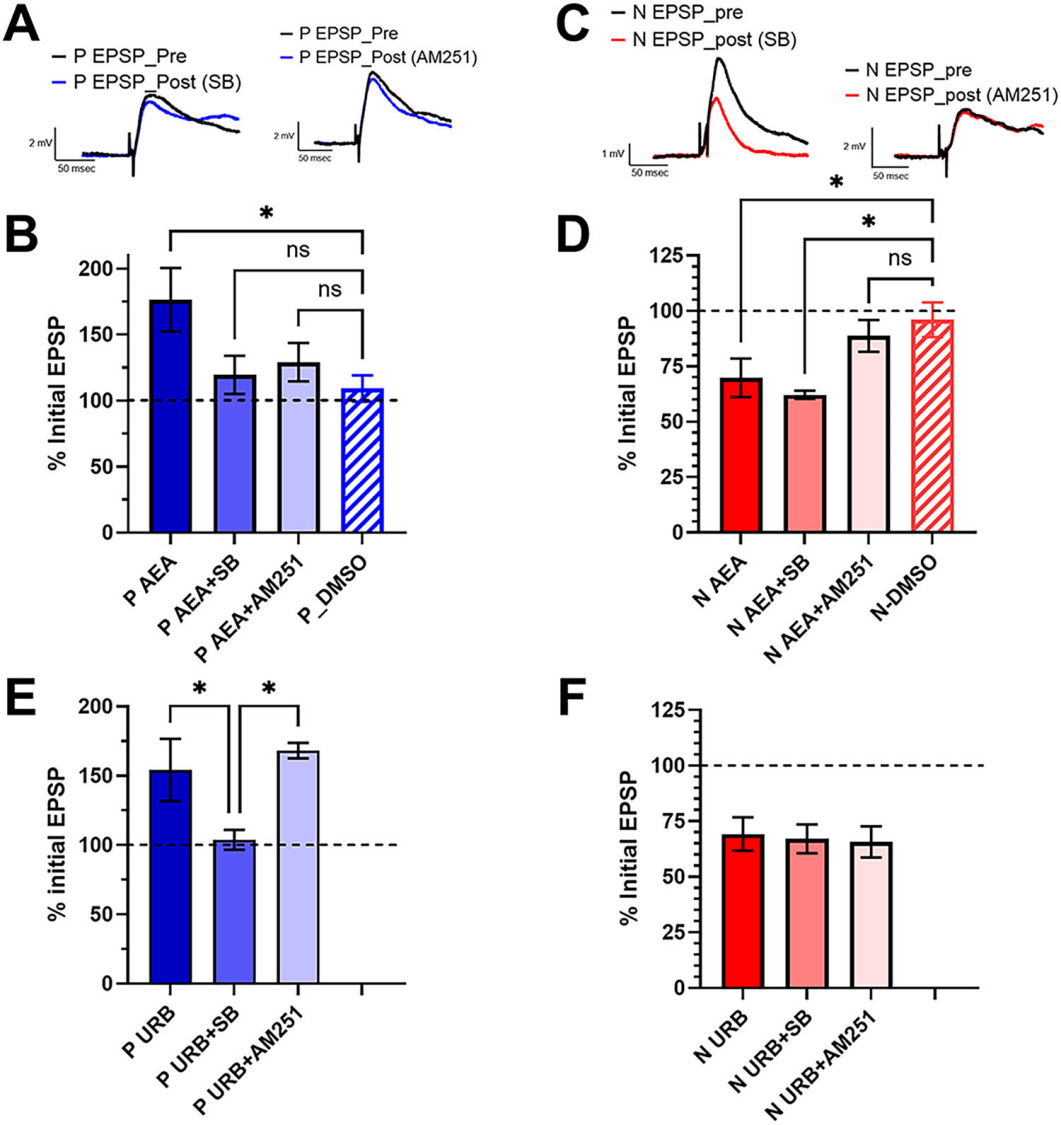
Pharmacology of AEA- and URB597-elcited potentiation and depression. ***A***, Sample P-AP synapse EPSP traces comparing pretest recordings in saline (P EPSP_Pre) and posttest recordings following AEA + SB366791 [P EPSP_Post (SB)] or AEA + AM251 [P EPSP_Post (AM251)]. ***B***, Comparison of the effects of AEA (*n* = 6), AEA + SB (*n* = 5), AEA + AM251 (*n* = 8), and 0.001% DMSO (*n* = 7) on P-AP synapse. ***C***, Sample N-AP synapse EPSP traces comparing pretest recordings in saline (N EPSP_Pre) and posttest recordings following AEA + SB366791 [N EPSP_Post(SB)] or AEA + AM251 [N EPSP_Post(AM251)]. ***D***, Comparison of effects of AEA (*n* = 8), AEA + SB (*n* = 6), AEA + AM251 (*n* = 7), and 0.001% DMSO (*N* = 7) on N-AP synapse. ***E***, Comparison of the effects of URB597 (P URB, *n* = 6), URB597 + SB366791 (P URB + SB, *n* = 8), and URB597 + AM251 (P URB + AM251, *n* = 5) on P-AP synapses. ***F***, Comparison of the effects of URB597 (N URB, *n* = 9), URB597 + SB366791 (N URB + SB, *n* = 4), and URB597 + AM251 (N URB + AM251, *n* = 4) on N-AP synapses. * indicates *p* ≤ 0.05.

Next, the effects of the TRPV1 inhibitor SB366791 and the CB1R inverse agonist, AM251, were examined. SB366791 has been previously shown to block eCB-LTD in N synapses and eCB-LTP in P synapses (Yuan and Burrell, 2010; Wang and Burrell, 2016). AM251 has been shown to block eCB-LTD in N synapses (Yuan and Burrell, 2010), but has not been tested in P-AP synapses. In P-AP synapses, potentiation by 10 µM AEA was blocked by both 10 µM SB366791 and 10 µM AM251 (Fig. 6*A*,*B*). A one-way ANOVA detected a significant effect of drug treatment (*F*_(3,22)_ = 3.18, *p* < 0.05), with only the AEA-treated ganglia being significantly different from the 0.001% DMSO control group (*p* < 0.05). In N-AP synapses, AEA-induced depression was blocked by AM251, but not by SB366791 (Fig. 6*C*,*D*). One-way ANOVA detected a significant effect of drug treatment (*F*_(3,24)_ = 4.47, *p* < 0.05) with both the AEA and AEA + SB groups significantly different from the 0.001% DMSO control group (*p* < 0.05 for both).

The effects of these two inhibitors on URB597-mediated synaptic changes differed from those produced by AEA. Potentiation of P-AP synapses by URB597 was blocked by SB366791, but not by 10 µM AM251 (Fig. 6*E*). A one-way ANOVA confirmed a statistically significant effects of SB366791 compared with URB597 (*F*_(2,16)_ = 6.64, *p* < 0.01, post hoc *p* < 0.05). In the case of the N-AP synapses, surprisingly neither SB366791 nor AM251 blocked URB597-mediated depression (Fig. 6*F*). A one-way ANOVA confirmed no statistically significant differences between the three treatment groups (*F*_(2,17)_ = 1.50, *p* = 0.25).

## Discussion

Attempts to develop cannabinoid-based therapies to treat pain have included activation of cannabinoid receptors and increasing levels of AEA or 2-AG via inhibition of FAAH or MAGL, respectively. Unfortunately, clear evidence that cannabinoid-based therapies are effective in treating pain is lacking (De Vita et al., 2018; Fisher et al., 2020; Mun et al., 2020; Finn et al., 2021; Hsu et al., 2025). One potential reason for this is that the endocannabinoid system can have pro- and anti-nociceptive effects as observed in both animal (Strangman et al., 1998; Hohmann et al., 2005; Liu and Walker, 2006; Pernia-Andrade et al., 2009; Bosshard et al., 2012; Ueda et al., 2014; Carey et al., 2016; Mascarenhas et al., 2017) and human studies (Christie and Mallet, 2009; Walter et al., 2015; Arout et al., 2022).

AEA pro- and anti-nociceptive effects have been observed following direct application of AEA, FAAH inhibitors to raise endogenous AEA levels, and application of AEA precursors (Morisset et al., 2001; Jennings et al., 2003; Kawahara et al., 2011; Starowicz et al., 2012, 2013; Carey et al., 2016; Mascarenhas et al., 2017; Nerandzic et al., 2018; Pontearso et al., 2024; Bhattacharyya et al., 2025). Pro- and anti-nociceptive effects of AEA have been ascribed to high versus low concentrations of AEA, respectively, possibly due to different concentrations preferentially activating CB1 versus TRPV1 (Morisset et al., 2001; Maione et al., 2006; Luchicchi and Pistis, 2012; Bhattacharyya et al., 2025). Dual effects of AEA may also depend on other factors, e.g., during inflammatory pain (Evans et al., 2004; Kawahara et al., 2011; Carey et al., 2016; Nerandzic et al., 2018; Pontearso et al., 2024).

The present study examined whether potentiation of non-nociceptive P-AP synapses and depression of nociceptive N-AP synapses were elicited by different AEA concentrations. Dual intracellular recordings of identifiable pre- and postsynaptic neurons were used so it was possible to selectively stimulate and measure nociceptive versus non-nociceptive evoked EPSPs. This is in contrast to many experiments on afferent transmission in the spinal cord that rely on spontaneous synaptic transmission where the identity of the presynaptic input is unknown. These experiments also examined the persistent effects of AEA or URB597 by recording from synapses tens of minutes after treatment, rather than testing synapses while drugs were still present. Finally, this study compared the effects of increasing concentrations of AEA and the FAAH inhibitor, URB597. This is significant because a FAAH inhibition has been one approach for developing a cannabinoid-based analgesic. An example is the FAAH inhibitor, PF-04457845, that exhibited clear analgesic effects in rodents but failed at the clinical trial level (Ahn et al., 2011; Huggins et al., 2012).

Interestingly, these approaches yielded two distinct sets of results. Direct application of AEA produced eCB-LTD in nociceptive N-AP synapses and potentiation in non-nociceptive P-AP synapses over the same concentration range. However, when URB597 was used, eCB-LTD in N-AP synapses was observed at much lower concentrations compared with potentiation in P-AP synapses. Furthermore, when a pattern of afferent activity that does not normally produce eCB-LTP in P-AP synapses (T-LFS) was used in combination with a subthreshold concentration of URB597, potentiation of the P-AP synapses now was observed. It appears that while this concentration of URB597 could not affect the P-AP synapses, it was able to “boost” endogenous AEA levels to a point where T-LFS could now elicit potentiation. The observed synaptic potentiation and depression do not appear to be due to changes in the intrinsic electrical properties of the postsynaptic AP cell given that no IR changes were observed. It is possible that there are localized changes in the neuropil that cannot be easily resolved by whole-cell measures of IR. However, PPF measurements of both the P-AP and N-AP synapses indicated a presynaptic locus consistent with changes in neurotransmitter release. Therefore, observed changes in EPSP amplitude appear to be primarily due to modulation of synaptic transmission, although contributions of localized changes in excitability cannot be excluded.

These findings suggest that while P-AP and N-AP synapses have intrinsically the same sensitivity to AEA, there are still functional differences in terms of how readily endogenous AEA can modulate either synapse. One potential explanation is that when FAAH inhibition is used to raise AEA levels, the AEA source that modulates N-AP synapses has a greater capacity for AEA synthesis relative to the source that modulates P-AP synapses. This could be due greater levels of NAPE-PLD in the source neuron where AEA is synthesized and released onto the N-AP synapse. Spatial factors may also play a role, with the N-AP synapses being potentially closer to the AEA source than the P-AP synapses (or the inhibitory neurons that mediate disinhibition of the P-AP synapses). In mammals endocannabinoid release is localized, potentially creating a spatial limit of what synaptic circuits are modulated (Dong et al., 2022). If this is the case in *Hirudo*, then local AEA concentration gradients could account for the observed differences between the N-AP and P-AP synapses. Presumably, exogenous application of AEA to the entire ganglion effectively bypasses these differences in the AEA sources. It is unlikely that the differences in AEA versus URB597 effects are due to differences in the diffusion rate for each drug since the treatment durations were the same, the ganglion sheathe was intact for all experiments, and both drugs have similar molecular weights (347.5 and 338.4 g/mol, respectively).

In the present study, the TRPV1 inhibitor, SB366791, did block the AEA-induced potentiation of P-AP synapses, consistent with previous studies in *Hirudo* in which this inhibitor blocked AEA-induced depression of tonic inhibition to the P cell that ultimately produced P synapse potentiation (Wang and Burrell, 2016; Paulsen and Burrell, 2022). That SB366791 did not block depression of N-AP synapses was somewhat surprising given that SB366791 has been shown to block eCB-LTD in N-AP synapses (Yuan and Burrell, 2010). TRP channels, including TRPV, are well represented in invertebrates (Vriens et al., 2004; Damann et al., 2008). No direct homologs of TRPV1 have been reported in invertebrates, but AEA- and 2-AG-mediated modulation of behavior and physiology via TRPV signaling (e.g., sensitive to capsaicin or SB366791) has been observed in planaria, mollusks, and *Caenorhabditis elegans* (Oakes et al., 2019; Sabry et al., 2019; Mosca et al., 2021; Abdollahi et al., 2024). This suggests that TRPV channels acting as endocannabinoid receptors are conserved between vertebrates and invertebrates. A TRPV-encoding gene has been sequenced and identified via single-cell PCR in the *Hirudo* N cells (Heath-Heckman et al., 2021). Furthermore, capsaicin depresses N synapses and potentiates P synapses, and these capsaicin effects were blocked by SB366791 (Yuan and Burrell, 2010; Wang and Burrell, 2016).

The CB1 inhibitor AM251 did block the effects of exogenous AEA on the P-AP and N-AP synapses. It should be noted, however, that whether *Hirudo* possess a homolog to CB1/2 receptors is unclear. Several studies report the efficacy of CB1 agonists (e.g., CPP55,940 and WIN55,212-2) and antagonists (e.g., AM251 and rimonabant) in the non-bilaterian invertebrate *Hydra*, and in protostome invertebrates, e.g., *Hirudo*, *Drosophila*, *C. elegans*, and multiple species of mollusk (De Petrocellis et al., 1999; McPartland et al., 2006; Lemak et al., 2007; Li and Burrell, 2009, 2010; Yuan and Burrell, 2010, 2012; Reis Rodrigues et al., 2016; Sunada et al., 2017; He et al., 2021). However, past screenings of complete *Drosophila* and *C. elegans* genomes found no evidence of homologs to the vertebrate CB1/2 receptors, although a potential ancestral CB receptor homolog has been identified in the pre-chordate deuterostome invertebrate *Ciona* (Egertova and Elphick, 2007; McPartland et al., 2007; Elphick, 2012). Consequently, it is unclear what receptor CB1 receptor agonists and antagonists are acting on in many invertebrates. Recently, a metabotropic endocannabinoid receptor has been identified in *C. elegans*, NPR-19, that has been proposed to be an ortholog of the vertebrate CB1/2 receptor (Pastuhov et al., 2016). NPR-19 responds to AEA, 2-AG, the phytocannabinoid tetrahydrocannabinol (THC), and the synthetic CB1 agonist arachidonyl-2′-chloroethylamide (ACEA; Oakes et al., 2017; Levichev et al., 2023; Boujenoui et al., 2024; Kim et al., 2024). Furthermore, the behavioral effects of NPR-19 loss-of-function mutations or knockdown in *C. elegans* can be rescued by expression of human CB1. To date, no orthologs of NPR-19 have been discovered in other protostome invertebrates.

Surprisingly, the receptor pharmacology of synaptic effects following URB597 treatment were not consistent with those of AEA. For the P-AP synapse, the TRPV1 inhibitor SB366791 still blocked potentiation following URB597 treatment, but AM251 was not ineffective. For the N-AP synapses, neither SB366791 nor AM251 prevented depression following URB597 treatment. One explanation for these results is that URB597 produces effects independent of raising AEA levels. Pharmacological inhibition or genetic knock-out of FAAH increases levels of other lipids, e.g., *N*-palmitoyl ethanolamine (PEA) and *N*-oleoyl ethanolamine (OEA; Ahn et al., 2011; Huggins et al., 2012; Carey et al., 2016; Leishman et al., 2016). The FAAH inhibitor PF-04457845 increased concentrations of OEA and PEA in both rodents and humans, in addition to increases in AEA (Ahn et al., 2011; Huggins et al., 2012). Furthermore, the analgesic effects of PF-04458845 in rodents were only partially blocked by CB1 or CB2 receptor inhibitors (Ahn et al., 2011). It is possible that FAAH inhibition in *Hirudo* produces similar increases in signaling lipids that produce effects independently of CB1 or CB2 receptors (Leishman et al., 2016).

Prior studies using *Hirudo* have reported endocannabinoid-mediated depression of N-to-L motor neuron (N-L) synapses accompanied by depression of withdrawal reflexes elicited by the same N cell in semi-intact preparations (Yuan and Burrell, 2013). Similar observations were made regarding endocannabinoid-mediated potentiation of P-to-L (P-L) synapses and sensitization of P cell-elicited withdrawal (Wang and Burrell, 2018). Although these studies utilized a different postsynaptic neuron than the current study, to date no differences have been observed in properties of endocannabinoid modulation P and N onto L motor neuron versus the AP neuron (Yuan and Burrell, 2010; Higgins et al., 2013; Wang and Burrell, 2016; Franzen et al., 2023).

The results of this study contribute to the collective evidence that the endocannabinoid system is utilized in distinct signaling pathways that mediate pro- versus anti-nociceptive effects. The problem is that most if not all of the cannabinoid-based therapies tested to date are likely activating both pro- and anti-nociceptive signaling pathways simultaneously, leading to no actual reduction in pain. This may explain why clinical studies using phytocannabinoids, synthetic activators of CB1, or FAAH inhibitors were no more effective than placebos (Huggins et al., 2012; Dieterle et al., 2022; Schneider et al., 2022; Zubcevic et al., 2023). There are also human subject studies in which cannabinoid treatment actually worsened pain (Beaulieu, 2006; Kraft et al., 2008; Walter et al., 2015; Arout et al., 2022).

In conclusion, while the present study has shown that nociceptive and non-nociceptive synapses are equally sensitive to AEA that is directly applied, endogenously released AEA appears to preferentially elicit depression in nociceptive synapses relative to potentiation in non-nociceptive synapses. Experiments are currently underway to examine concentration-dependent effects of 2-AG on nociceptive and non-nociceptive synapses in *Hirudo*. Given the apparent conservation of endocannabinoid neuromodulatory function, these findings may help to guide future experiments aimed at developing effective cannabinoid-based analgesics.

## Synthesis

Reviewing Editor: Mark Sheffield, University of Chicago Division of the Biological Sciences Decisions are customarily a result of the Reviewing Editor and the peer reviewers coming together and discussing their recommendations until a consensus is reached. When revisions are invited, a fact-based synthesis statement explaining their decision and outlining what is needed to prepare a revision will be listed below. The following reviewer(s) agreed to reveal their identity: Lidia Szczupak.

The manuscript leverages the experimentally accessible leech ganglion to investigate an important and unresolved question in cannabinoid biology: why endocannabinoids produce both inhibitory and disinhibitory effects within nociceptive pathways, yet have not translated into reliable analgesics in mammals. The preparation is well suited for isolating synaptic mechanisms, the experiments are carefully designed, and the electrophysiological data are clearly presented. The finding that exogenous AEA does not differentially affect inhibitory versus disinhibitory pathways by concentration, while pharmacological elevation of endogenous endocannabinoids reveals apparent differences in sensitivity, addresses a mechanistic hypothesis of genuine interest. At the same time, several interpretive and conceptual issues limit the strength of the conclusions as currently framed.

A central concern is that some interpretations extend beyond what the data can firmly establish. The differential effects observed with URB597 are attributed to distinct sensitivities of N and P synapses to endogenous AEA, but alternative explanations remain plausible and are not fully explored. These include differences in drug diffusion or kinetics between AEA and URB597, insufficient equilibration time for URB597 to elevate endocannabinoids uniformly, or threshold effects within inhibitory interneurons that mask disinhibition until a critical level is reached. In addition, spatial factors may play a role: if endogenous AEA release sites are closer to N than P terminals, local concentration gradients rather than intrinsic synaptic sensitivity could account for the observed differences. The electrophysiological data themselves (e.g., differences in EPSP kinetics) may contain clues about synapse location within the neuropil that could inform this issue, but such analyses are not currently presented.

Relatedly, several mechanistic aspects require clarification. It is not fully established how endogenous AEA reaches synaptic sites within the ganglion, nor whether the ganglion was desheathed, both of which affect diffusion and interpretation. The possibility that AEA or URB597 alters postsynaptic input resistance, and thereby synaptic amplitude, should also be examined, as changes in intrinsic membrane properties could contribute to the observed effects. Additional methodological clarifications (e.g., validation of URB597 effects in the leech system, correction of statistical reporting, and clarification of paired-pulse data) would further strengthen rigor and transparency.

A second major theme concerns conceptual framing. The manuscript frequently refers to "pro-nociceptive" and "anti-nociceptive" effects when describing potentiation of P synapses and depression of N synapses. However, the functional interpretation of P synapses as pro-nociceptive is not sufficiently justified in the current presentation. It remains unclear whether the goal is to distinguish effects on nociceptive versus non-nociceptive pathways, or to make broader claims about behavioral consequences. Greater precision in terminology, and careful justification of any functional assignments, would avoid overinterpretation. Similarly, the generic reference to "P" and "N" synapses without specifying the postsynaptic AP target risks implying generality across all targets. If the AP neuron is intended to be representative, this assumption should be explicitly justified; otherwise, referring to P-AP and N-AP synapses would more accurately describe the experimental system without diminishing its significance.

The manuscript also touches on translational implications. While the parallels between leech and mammalian endocannabinoid modulation are striking and potentially informative, conclusions suggesting that the leech system reveals fundamental features "necessary" for developing effective human analgesics are viewed as too strong. Given the deep evolutionary divergence between taxa, a more balanced framing would emphasize the apparent conservation of functional motifs and position the findings as generating hypotheses for mammalian systems rather than directly prescribing translational solutions.

Several additional clarifications would improve precision and accessibility. The Introduction should be written in the past tense to reflect completed experiments. Claims regarding TRPV1 should explicitly note its absence in leech while acknowledging the presence of other TRP channels. Taxonomic statements (e.g., Hydra) should be corrected. Ambiguities in the Abstract (e.g., wording around "produced" and the effects of URB597 on P synapses) should be resolved. Statistical reporting errors and missing references need correction.
