# Supplementary figures and images for: Role of Concentration in Opposing Effects of Anandamide on Nociceptive Synapses versus Non-nociceptive Synapses
Source: eNeuro. 2026 Apr 30;13(5):ENEURO.0480-25.2026. doi: 10.1523/ENEURO.0480-25.2026 (PMC13159970; doi:10.1523/ENEURO.0480-25.2026)

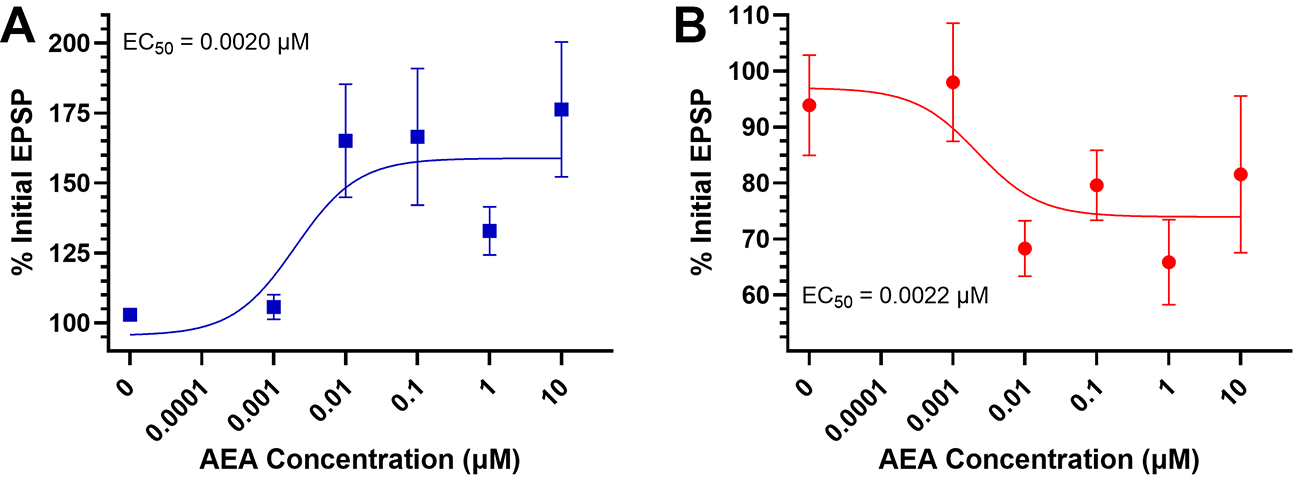

Supplement: Figure 3-1 — Comparison of concentration profiles for AEA. (A) P-AP synapse. (B) N-AP synapse. Download Figure 3-1, TIF file. [file eneuro-13-ENEURO.0480-25.2026-s002.tif]

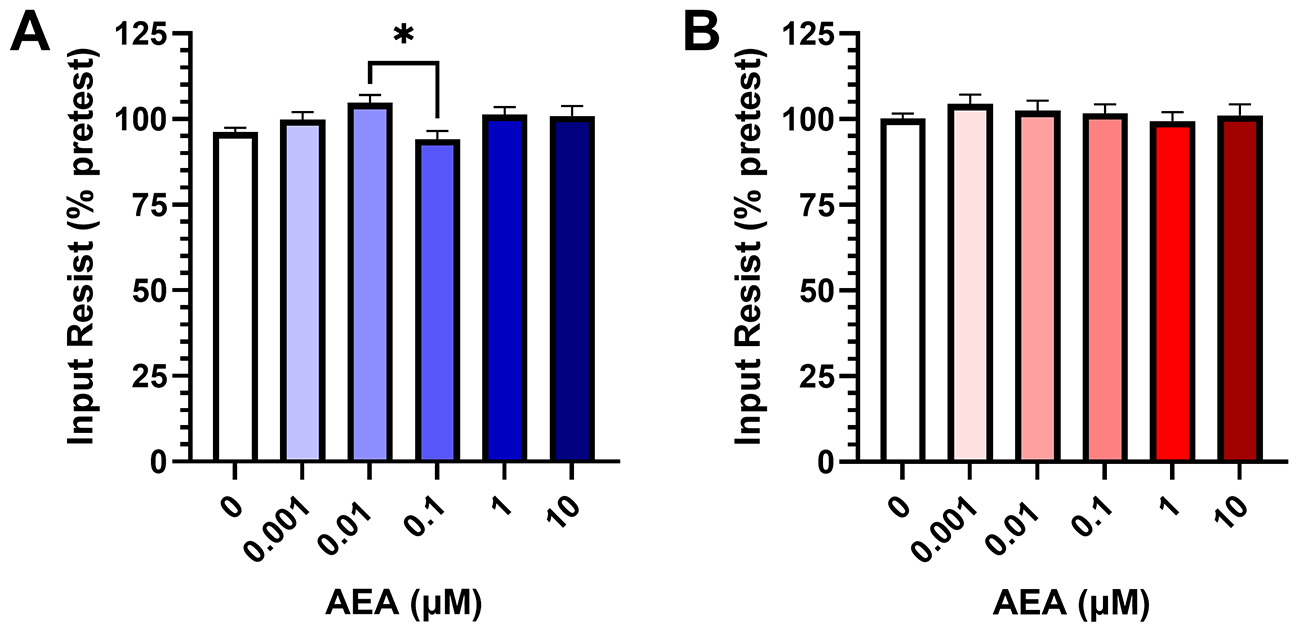

Supplement: Figure 3-2 — Effects of AEA on postsynaptic (AP cell) input resistance (IR). Data is expressed as a percent of the initial pretest level (100[posttest IR/pretest IR]). Effects of increasing AEA concentrations from the P-AP (A) and N-AP (B) synapses. In the P-AP synapses, none of the AEA-treated synapses were different from the vehicle control group (0 μM), although there was a statistically significant difference in IR between the 0.01 and 0.1 μM groups (p<0.05). In the N-AP synapses, none of the AEA-treated synapses were different from the vehicle control group. Download Figure 3-2, TIF file. [file eneuro-13-ENEURO.0480-25.2026-s003.tif]

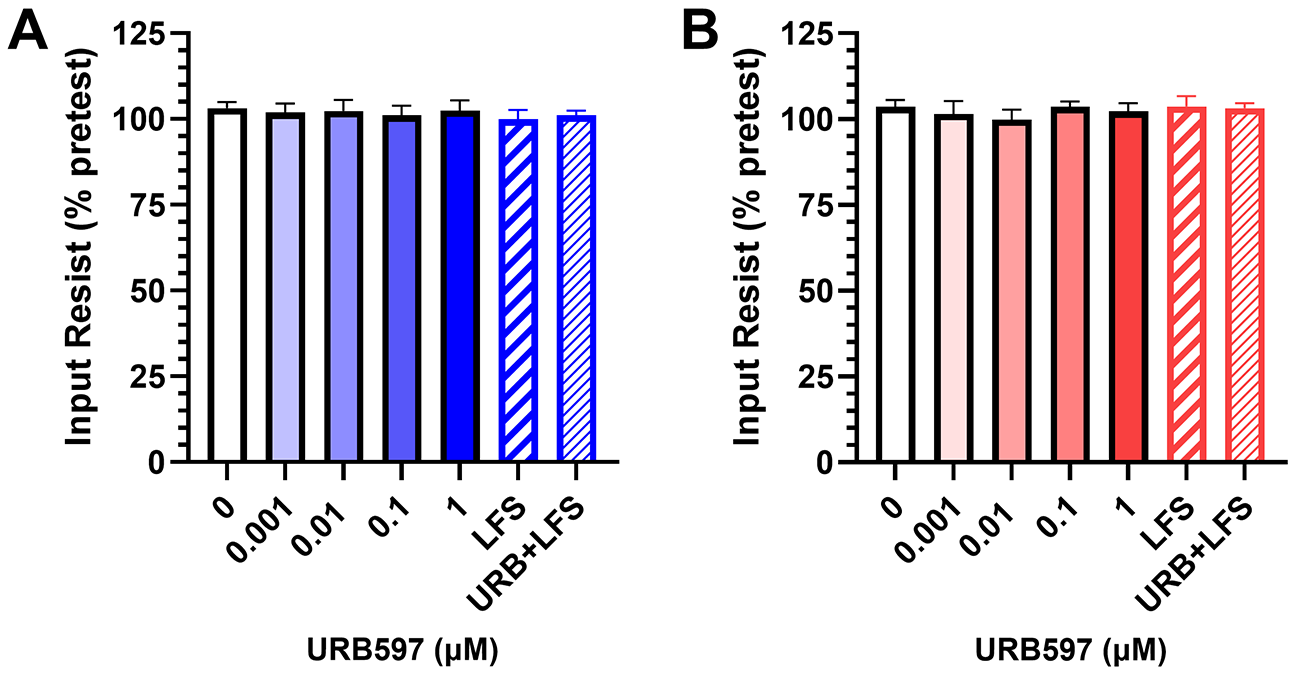

Supplement: Figure 5-1 — Effects of URB597 and T-LFS on postsynaptic IR. Increasing AEA concentrations are represented by solid fills. Effects of LFS and URB+LFS treatments are shown as diagonal fill patterns. None of these treatments exhibited an effect on input resistance in P-AP (A) and N-AP (B) synapses. Download Figure 5-1, TIF file. [file eneuro-13-ENEURO.0480-25.2026-s004.tif]
